# Precise Temporal Modulation in the Response of the SOS DNA Repair Network in Individual Bacteria

**DOI:** 10.1371/journal.pbio.0030238

**Published:** 2005-06-21

**Authors:** Nir Friedman, Shuki Vardi, Michal Ronen, Uri Alon, Joel Stavans

**Affiliations:** **1** Department of Physics of Complex Systems, The Weizmann Institute of Science, Rehovot, Israel,; **2** Department of Molecular Cell Biology, The Weizmann Institute of Science, Rehovot, Israel; Institut National de la Recherche AgronomiqueFrance

## Abstract

The SOS genetic network is responsible for the repair/bypass of DNA damage in bacterial cells. While the initial stages of the response have been well characterized, less is known about the dynamics of the response after induction and its shutoff. To address this, we followed the response of the SOS network in living individual *Escherichia coli* cells. The promoter activity (PA) of SOS genes was monitored using fluorescent protein-promoter fusions, with high temporal resolution, after ultraviolet irradiation activation. We find a temporal pattern of discrete activity peaks masked in studies of cell populations. The number of peaks increases, while their amplitude reaches saturation, as the damage level is increased. Peak timing is highly precise from cell to cell and is independent of the stage in the cell cycle at the time of damage. Evidence is presented for the involvement of the *umuDC* operon in maintaining the pattern of PA and its temporal precision, providing further evidence for the role UmuD cleavage plays in effecting a timed pause during the SOS response, as previously proposed. The modulations in PA we observe share many features in common with the oscillatory behavior recently observed in a mammalian DNA damage response. Our results, which reveal a hitherto unknown modulation of the SOS response, underscore the importance of carrying out dynamic measurements at the level of individual living cells in order to unravel how a natural genetic network operates at the systems level.

## Abstract 2

Oscillations in transcriptional activity in the network responsible for controlling DNA damage are monitored with GFP- promoter fusions in individual *E. coli* cells.

## Introduction

The SOS genetic network [1–3] includes more than 30 genes in *Escherichia coli* [4,5] that carry out diverse functions in response to DNA damage, including nucleotide excision repair, translesion DNA replication, homologous recombination, and cell division arrest. A vast amount of biochemical, genetic and structural data are available on the various components of the network and the interactions between them, which makes the system a paradigm for studying and modeling regulation of DNA repair [6–9]. The network is controlled by the LexA repressor, which downregulates itself and the expression of the other SOS genes. Following DNA damage, a RecA nucleoprotein filament is formed along stretches of single-stranded DNA (ssDNA) near arrested replication forks. This RecA filament promotes autocleavage of the LexA repressor, leading to induction of the response [6]. While the initial stages of the SOS response have been well characterized, the temporal coordination of events during the response and its shutoff are poorly understood.

Previous experiments investigated the dynamics of the response at the population level, using DNA microarrays [5], or a fluorescent protein as a reporter for promoter activity (PA) [10]. Those studies revealed an increase in the level of transcripts and of PA of SOS genes, respectively, after induction by ultraviolet (UV) irradiation. This was followed by a decrease in the activation of these genes, presumably when DNA damage was repaired and the system was shut off. Such a single-peaked response is expected from a simple model of the network, in which repression of transcription by LexA is the only regulation mechanism.

Measurements performed over a population of cells might be limited in their ability to accurately describe network responses in the case of a nonhomogenous population, or an unsynchronized response. Examples include systems that show an all-or-none response [11] that is averaged out at the population level, steep response curves [12], or asynchronous oscillations [13] that are smeared out in ensemble measurements. Thus, a full understanding of a network's responses and the ability to understand them using computational models require experimental knowledge about the dynamics in individual cells.

In this study, the dynamics of SOS response was investigated at high temporal resolution in individual living cells, using the green fluorescent protein (GFP) as a reporter for PA. Contrary to the single-peaked response observed in population studies, our measurements reveal that the response is highly structured, with precise temporal modulations of gene expression levels.

## Results

### The SOS Response Exhibits Discrete Activation Peaks

To measure the dynamics of the SOS response at the level of individual cells, the activity of LexA-repressed promoters *(recA, lexA,* and *umuDC)* was monitored using low-copy reporter plasmids in which the promoter under investigation was fused to a *gfp* gene whose product becomes fluorescent within minutes of transcription initiation (*gfpmut2* [14]). The accumulation of GFP in a cell is proportional to the rate of transcript production from the promoter [10,15]. We used time-lapse fluorescence microscopy to measure the fluorescence intensity and size of bacteria containing the reporter plasmids over 150 min following DNA-damaging UV irradiation, at a 2-min temporal resolution. Typical snapshots obtained at two times during the response of a number of cells after a 10 J/m^2^ UV dose are shown in Figure 1A and 1B.

**Figure 1:**
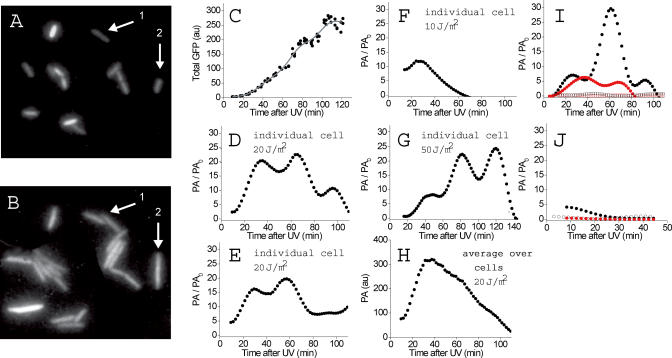
Dynamics of the SOS Response Observed in Individual Cells (A and B) Snapshots from a time-lapse movie monitoring the fluorescence of live AB1157 *E. coli* cells taken (A) 8 and (B) 70 min after irradiation with a UV dose of 10 J/m^2^. Cells are expressing GFP as a reporter for the *recA* PA [10]. Some cells grow and undergo cell division (e.g., cell #1), while others exhibit filamentation (e.g., cell #2), as a consequence of DNA damage. The exposure time corresponding to (A) is ten times that for (B). (C) Total GFP produced from the *recA* promoter as a function of time, measured in an individual cell irradiated at 20 J/m^2^. The full line corresponds to the data after filtration, which is used to compute the PA. (D) PA/PA_0_ as a function of time for the same cell as in (C). (E–G) Normalized *recA* promoter activity PA/PA_0_ as a function of time for cells irradiated at 20, 10, and 50 J/m^2^, respectively. (H) Average *recA* PA over all 23 cells in an experiment at 20 J/m^2^. (I) *recA* PA/PA_0_ for two noninducible LexA(Ind^−^) cells (empty circles), and for two isogenic LexA^+^ cells (full circles), all irradiated at 20 J/m^2^. (J) *recA* PA/PA_0_ from an unirradiated cell (empty circles), and *lacZ* PA/PA_0_ from two cells irradiated at 20 J/m^2^ (full circles).

The length, *L*(*t*), and average fluorescence intensity, *I*(*t*), of each cell in the field of view were measured for each image, and the product *I*(*t*)*L*(*t*), proportional to the total amount of GFP in a given cell at time *t* was calculated, as illustrated in Figure 1C. The PA was then computed as the rate of change of fluorescence per unit cell size: (see Materials and Methods). The normalized PA (PA/PA_0_) of the *recA* promoter as a function of time, in representative cells irradiated with different doses of UV radiation, is plotted in Figure 1D–1G (PA_0_ is the average PA of uninduced cells; see Materials and Methods). We find that the measured response of individual cells is highly structured. Up to three peaks in PA are typically observed within the duration of the experiments, with the typical number of peaks increasing with damage level. In contrast to the modulations found in cells following DNA damage, unirradiated cells exhibited a constant PA, close to the uninduced level, as shown in Figure 1J. A non-SOS promoter *(lacZ)* showed a low and constant or decreasing PA following UV damage (Figure 1J). As another control, we monitored the response of noninducible LexA(Ind^–^) cells (KY703), which did not show any response after damage (Figure 1I). The isogenic LexA+ strain (KY700; see Materials and Methods) responded in a similar way to the AB1157 strain, as shown in Figure 1I.

### Response Peaks Exhibit Precise Timing

To evaluate the distribution of peak parameters among the cell population, we plot in Figure 2A and 2B the amplitude and time of the *recA* PA peaks for each and every cell in the experiments, for two different UV doses. We find that the data form distinct clusters, characterized by narrow variability in peak timing but larger variability in PA peak values. Note that at 50 J/m^2^, the peaks appear at later times than at 20 J/m^2^. To further characterize the timing of the peaks, we plot in Figure 2C and 2D the corresponding histograms of peak times. The histograms exhibit three narrow peaks (standard deviation/mean less than 10%; see also Table 1) showing that peak timing is quite accurate among different cells under the same UV dose. In contrast, the variability of peak amplitude is larger (standard deviation/mean greater than 25%; see also Table 1). Some of the measured variance in PA timing and amplitude stems from cell-to-cell variability in the copy number of the plasmid-borne reporters [16]. Thus, these values serve as an upper limit, and actual variance for the chromosomal promoter may by even lower. Discrete activation peaks were observed also in the PA of the *lexA* and *umuDC* promoters (Figure 2E–2H). While no significant difference in peak timing between the *lexA* and *recA* promoters was observed (Figure 2E and 2F), peaks in the activity of the *umuDC* promoter were delayed by 7–10 min (Figure 2G–2H). Similar delays in the decay of activity of different promoters of the SOS network have been observed in studies of populations [10].

**Figure 2:**
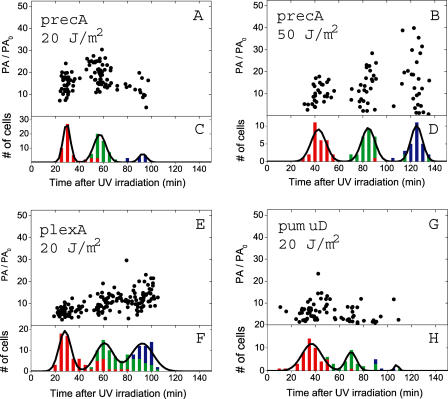
Quantitative Analysis of the Oscillatory Behavior: Distributions of Peaks' Amplitude and Time Normalized amplitudes of the peaks in *recA* PA are plotted as function of peak time for individual cells irradiated with a UV dose of (A) 20 J/m^2^, (B) 50 J/m^2^. Each point corresponds to one peak in an individual cell. The three clusters in (A) (total of 51 cells) are centered at *T*_1_ = 29 ± 3 min, *T*_2_ = 57 ± 5 min, and *T*_3_ = 93 ± 4 min (mean ± standard deviation). The average normalized promoter activities corresponding to these three clusters are: PA_1_ = 15 ± 4, PA_2_ = 19 ± 5, and PA_3_ = 13 ± 4 in units of PA_0_. (C and D) Histograms of peak times corresponding to (A) and (B), ranking each peak by its order of appearance: red: first peak in a trace of PA(*t*) of an individual bacteria; green: a second peak in its trace; blue: a third peak in its trace. Black lines: fits to the histograms with a sum of Gaussians. (E) Peak *lexA* PA as function of peak time for individual cells and (F) its corresponding histogram. (G) Peak *umuDC* PA as function of peak time for individual cells and (H) its corresponding histogram. Cells in the experiments (E) and (G) were irradiated with a UV dose of 20 J/m^2^.

**Table 1:**
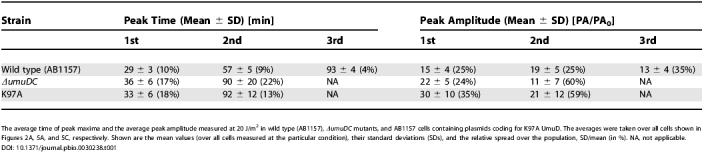
Mean Values and Standard Deviations for the Peaks' Time and Amplitude

Given the precision observed in peak timings, one may wonder why temporal modulations have not been observed in experiments over cell populations. To address this, we computed the PA averaged over all individual cells in an experimental run. As Figure 1H illustrates, peaks are washed out due to their slight missynchronization, highlighting the importance of carrying out these experiments in individual cells. Measurements of the response in cell cultures [10] show a single activation peak at about 30 min after irradiation, except for high UV doses (greater than 40 J/m^2^), where another small peak was observed around 90 min.

### Peak Timing Correlates with the Cell Growth Rate but Not with the Stage in the Cell Cycle

Measuring the response in individual cells allows not only for the evaluation of distributions of response dynamics in a population, but also for the calculation of correlations between these dynamics and parameters related to cellular growth. We find that there is a lack of correlation between the peak time and the size of the cells at the time of irradiation, as is shown in Figure 3A. This indicates that peak timing is not synchronized with the bacterial cell cycle. The peak time is however correlated with the cell's growth rate (1*/T_D_*) after irradiation, as shown in Figure 3B (*T_D_* is the time it takes for a cell's length, or for the sum of lengths of its daughter cells, to double). The linear relation that exists between 1*/T*_1_ and *1/T_D_* (1/*T*_1_ **=** 1/*T_D_* **+** 1/τ) suggests that the peaks' timing is governed by the effective lifetime of a factor that is diluted by cell growth at a rate 1*/T_D_* and is degraded at a rate 1/τ = 1/68 min^–1^ [17], which is independent of cell growth rate and of UV dose. Note that the timings of the three peaks *T*_1_, *T*_2_, and *T*_3_ themselves are positively correlated (Figure 3C), even at the level of individual cells: cells in which *T*_1_ is larger than the average, tend to have a larger than average *T*_2_ and *T*_3_ as well (*T*_1_, *T*_2_, *T*_3_ correspond to the time of the first, second, and third peak, respectively, observed in the same bacteria). As for the peaks' amplitudes, no correlation was observed between PA*_i_*/PA_0_ and either the cell size at the time of irradiation or the cell growth rate.

**Figure 3:**
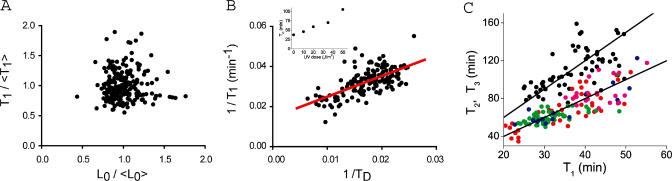
Correlations between the Peaks' Time and Bacterial Growth Parameters (A) Scatter plot of the time of the first peak, *T*_1_, (normalized by the population average value <*T*_1_>) vs. the length of the cell at the time of UV irradiation, *L*_0_ (normalized by the population average <*L*_0_>). All cells from all UV doses are included; each point represents an individual cell. No correlation between the two quantities is observed (correlation coefficient = 0.02, *p* = 0.82). (B) Scatter plot of 1/*T*_1_ as a function of the growth rate, 1/*T_D_,* of individual cells irrespective of dose. A significant correlation is observed (correlation coefficient = 0.66, *p* < 10^–4^). A linear fit to the data yields a slope of 1.0 ± 0.1. Inset: Cell doubling time *T_D_* grows monotonically as a function of UV dose. (C) Time of the second (*T*_2_) and third (*T*_3_) peaks is plotted against the time of appearance of the first peak (*T*_1_). Each point corresponds to an individual cell. The data for *T*_2_ correspond to 10 J/m^2^ (green), 20 J/m^2^ (red), 35 J/m^2^ (blue), and 50 J/m^2^ (magenta). The data for *T*_3_ are shown in black irrespective of radiation dose for the sake of clarity. Full lines, *T*_2_ = 2*T*_1_, and *T*_3_ = 3*T*_1_ are shown as a guide to the eye. Averaging over all experiments at all UV doses we obtain: <*T*_2_/*T*_1_> = 1.99 ± 0.02 (mean ± standard error, over 132 cells), whereas <*T*_3_/*T*_1_> = 2.99 ± 0.06 (over the 60 cells that show a third peak). Peak times in A, B, and C are from measurements performed with the *recA* promoter.

### The Number of Peaks Grows with UV Dose, Their Normalized Timing Is Constant, and Their Amplitude Saturates

Next, we analyzed the dependence of the response parameters on the UV dose. The number of peaks observed increases with the amount of damage (Figure 4A). At a UV dose of 10 J/m^2^, most cells (approximately 60%) show a single activation peak, while some show two and even three peaks (approximately 25% and 15%, respectively) with a relatively low amplitude (see below). At 50 J/m^2^, on the other hand, most cells (approximately 55%) show three peaks, whereas 35% show two peaks, and no more than 10% show only one peak. In contrast, the normalized timing of the peak maxima averaged over all cells, *<T_i_>/T* (*i* = 1, 2, 3; *T* = [1/*T_D_* + 1/τ]^–1^ ), is constant over the 10–50 J/m^2^ dose range (Figure 4B). The average amplitude of the peaks shows some dependence on the UV dose, but it becomes saturated at approximately 20 PA_0_ at doses above 20 J/m^2^ (Figure 4C). These observations indicate that the cells respond to increasing damage levels by increasing the number of activation cycles, rather than by increasing the amplitude of the response.

**Figure 4:**
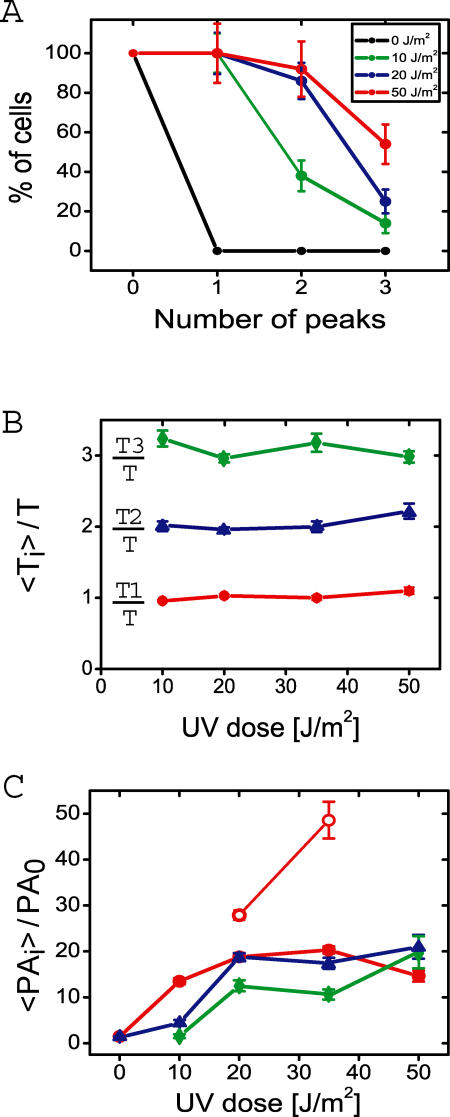
Dependence of Response Parameters on UV Dose (A) Percentage of cells exhibiting at least zero, one, two, or three peaks at different damage levels. (B) Dependence of the mean peak time <*T_i_*>, normalized by *T =* (1/*T_D_* + 1/τ)^–1^ (τ = 68 min), on UV dose. (C) Dependence of the mean normalized peak height <PA*_i_*>/PA_0_ on UV dose. Wild type (full symbols), *ΔumuDC* (empty symbols). Same color and symbol convention as in (B): <PA_1_>/PA_0_ (red circles), <PA_2_>/PA_0_ (blue triangles), and <PA_3_>/PA_0_ (green diamonds). In wild-type cells, the amplitude of peaks saturates at approximately 20 PA_0_ for UV doses greater than 20 J/m^2^. *ΔumuDC* cells do not show this saturation and reach much higher PA levels. Parameters in (A), (B), and (C) are from measurements performed with the *recA* promoter.

### The *umuDC* Operon Is Involved in Maintaining the Pattern of Activity Peaks and Its Precision

Since the modulations are observed in the activity of all three promoters investigated, it is likely that they represent modulations in the level of the master transcriptional regulator LexA. Thus, the behavior described above reveals the existence of another level of regulation of the SOS response, beyond the transcriptional control by LexA. What are the mechanism(s) underlying the precise temporal modulations of PA? It has been recently proposed that the products of the *umuDC* operon may act as a prokaryotic DNA damage checkpoint, effecting a timed pause in DNA replication [18], in addition to their role as an error-prone DNA polymerase (PolV) [19–22]. This has motivated our hypothesis that the products of *umuDC* may be involved in the mechanism behind the observed modulations.

To test this hypothesis, we repeated the measurements on a strain deleted for the *umuDC* operon *(ΔumuDC)* (Figure 5). We find that a number of important changes are observed in the response of this mutant, compared to the wild-type strain: first, most cells do not show the well-defined peak of PA around 60 min after irradiation. Instead, the prevailing pattern is a peak of PA around 30 min, followed by another peak appearing between 70 and 110 min, with a very large variance of cell-to-cell timing and amplitude (see Figure 5A and 5B; Table 1). Second, the amplitude of the first peak reaches higher levels relative to wild type (compare Figure 5A with Figure 2A) and does not show saturation with UV dose (see Figure 4C). Moreover, the amplitude and timing of the first peak are more correlated than in wild-type cells (Figure 5A). Thus if the first peak in a *ΔumuDC* cell appears later than the average, it will tend to have a higher amplitude, whereas in wild-type cells, no such correlation is observed. Third, peak time becomes independent of growth rate (Figure 5E).

**Figure 5:**
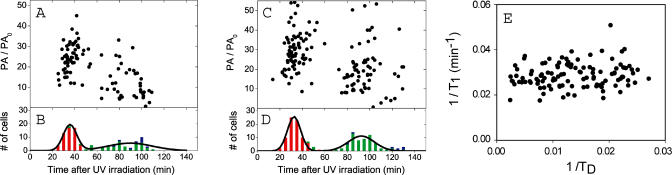
Effects of the *umuDC* Operon on the Temporal Modulation of Promoter Activity (A) Peak PA of the *recA* promoter as a function of peak time for individual *ΔumuDC* cells and (B) its corresponding histogram of the peak times. The amplitude and timing of the first peak are more correlated (correlation coefficient = 0.37, *p* = 0.002) than in wild-type cells (Figure 2A, correlation coefficient = 0.18, *p* = 0.13). (C) Peak PA of the *recA* promoter as a function of peak time for individual AB1157 cells transformed with a noncleavable *umuD* mutant gene (K97A) expressed from a plasmid, and (D) its corresponding histogram of the peak times. The experiments were carried out at 20 J/m^2^. (E) Scatter plot of 1/*T*_1_ as a function of the growth rate, 1/*T_D_,* of individual *ΔumuDC* cells irrespective of UV dose. In contrast with wild-type behavior (see Figure 3B), experiments with *ΔumuDC* mutants show that the timing of first peak maxima and cell growth rate 1/*T_D_* are poorly correlated (correlation coefficient = 0.15, *p* = 0.06, compared with a correlation coefficient = 0.66, *p* < 10^–4^ measured for AB1157 cells).

A further test of the involvement of *umuD* in setting the precision of the peak timing and its effect on the second peak is furnished by experiments with a dominant-negative, noncleavable *umuD* mutant gene (K97A), expressed from a plasmid in AB1157 cells (Figure 5C, 5D, and Table 1). As with *ΔumuDC,* the amplitude of the first peak does not saturate and increases considerably; the peak at 60 min completely disappears, and in its place a minimum in *recA* PA is observed. Furthermore, the peak centered at approximately 90 min appears with a high timing and amplitude variance among cells.

These observations indicate that the cleaved form UmuD′ is required for the reactivation of SOS PA at around 60 min after irradiation. It is interesting to note that the timing of reinitiation of DNA replication after damage reported in Opperman et al. [18] is similar to the timing of the observed second peak of the SOS response, and both require the existence of UmuD′.

## Discussion

The present findings show that the SOS response is highly structured, exhibiting discrete peaks in the PA of some of the genes in the network, peaks that appear with high temporal precision. Mutations in the *umuDC* genes affect the precision of these modulations.

A number of features of the response are important when evaluating possible mechanisms underlying the observed modulations. First, the observed similarity of the temporal patterns of activity of the three promoters studied suggests that the patterns are caused by modulation of LexA levels. This suggests that posttranscriptional regulation plays a role in regulating SOS dynamics. Second, the peak times, normalized by the average doubling time are independent of UV dose, and there is a lack of correlation between the peak time and the length of the bacteria at the time of irradiation. Thus, the normalized timing and phase of the modulations do not depend on factors such as the number of chromosomes (and hence the mean number of DNA lesions) at the time of irradiation. Third, peak timings exhibit a very low variability between cells, in spite of cell–cell variations in network component numbers (e.g., proteins), amount of damage, and the different growth stages at the time of irradiation.

Our results demonstrate that the products of the *umuDC* operon play an important role in generating the temporal modulation of activity, maintaining its temporal precision, and endowing the SOS response with “digital pulses” [23], whose number but not amplitude increases with damage. In the absence of *umuDC* the response is analog in nature in that (i) the amplitude of the first peak increases with damage level, whereas in wild-type cells it saturates, and (ii) the amplitude of the first peak and its timing are correlated, in contrast with the amplitude-independent peak timing characteristic of a digital response.

However, neither deletion of the *umuDC* operon nor the K97A dominant negative, non-cleavable umuD protein fully eliminates the modulations. Both mutants show peaks of activity at around 30 and 90 min after irradiation at 20 J/m^2^. Thus, other factors in the network of interactions between components of the SOS response must also play a role. The active RecA* nucleoprotein filament is known to interact with additional factors induced by the SOS response, which modulate its coprotease activity. These factors include LexA and products of the *umuDC* operon, as well as the RecX [24,25] and DinI [26,27] proteins and double-stranded DNA during homologous recombination [28]. These factors might play a role in the decrease of activity after the first and third peaks, which are less affected by the *umuDC* gene products.

In addition, the level of ssDNA in the cell changes during the response due to the progression of repair processes and the detection of new damage sites by propagating replication forks. The observed peak at 60 min may be an example of such a process, where the delayed translesion synthesis activity of PolV [29] causes formation of ssDNA in newly detected damage sites and, thus, reinitiation of the response. This explanation is in accord with the observed elimination of this peak in the ΔUmuDC and uncleavable UmuD mutants. This process can be more accurately timed and synchronized by the previously proposed role of uncleaved UmuD as a checkpoint inhibiting DNA synthesis after damage, and the timed release of this checkpoint by UmuD cleavage [29]. Other changes in activity may result from the encounter of persistent lesions during the next round of replication from OriC. This scenario is not favored by the observed lack of correlation between peak timing and the length of cells at the time of irradiation, unless there is a timed pause in initiation of replication from OriC after SOS induction. Such a pause was observed only at high levels of UV irradiation (greater than 60 J/m^2^) [30].

The present findings show that the progress of the response is accurately timed, irrespective of the level of the damage. The induction of the SOS response after irradiation inhibits DNA replication [31,32] and cell division [3], establishing a common reference time point for all cells, which are otherwise unsynchronized in their cell cycle. Thereafter, features of the SOS network, such as the different affinities of LexA to its binding sites on the different promoters, and critical events, such as UmuD cleavage, govern the temporal execution of the response and can lead to the synchronization.

Another possible mechanism behind the temporal modulation of PA is the existence of one or more negative feedback loops in the network. As mentioned above, factors such as DinI, RecX, the products of the *umuDC* operon, and double-stranded DNA during homologous recombination modulate the stability and coprotease activity of the nucleoprotein RecA filament. The increase in the concentration of these factors after SOS induction may lead to a decrease in the rate of LexA degradation, and consequently to a decrease in SOS induction levels, leading to a negative feedback. Any of these possibilities is likely to play a role in the reduction of PA after peaks. This type of negative feedback loops, particularly those with delays, such as transcription delays, can give rise to oscillatory behavior [33,34]. The nearly integer ratio (1:2:3) between the timing of the peaks T_1_:T_2_:T_3_ in our experiments may support this scenario (see Figure 3C and Table 1).

Further experiments with other mutants would be required in order to explore the full mechanism behind the observed modulations and to understand their role in the cellular response to DNA damage. Of particular interest are mutations of other genes that interact with RecA, such as DinI and RecX, and mutations in RecA that show selectivity for either LexA or UmuD cleavage [35].

Finally, we suggest that the SOS network displays a number of design features that may also occur in other repair systems. First, it *accurately times and synchronizes* the repair process, thus allowing cells to respond not only according to the current amount of damage but also according to the time elapsed since damage was detected. One such timing mechanism, namely UmuD cleavage and its role as a DNA replication checkpoint, allows for an interval during which precise repair is carried out, while DNA replication is arrested and the levels of other repair enzymes are rising. In addition, mutagenesis by PolV may be limited to a short time window, starting from UmuD cleavage and ending by UmuD′ proteolysis. Second, it affords *differential temporal activation* of various promoters by the modulations in the level of a common repressor [10]. Third, the SOS network includes mechanisms to *limit the response level,* thereby avoiding a response that is too high at early stages. The decrease of the response after each peak may allow the cell to evaluate whether damage still persists and permit rapid shutoff when repair has been accomplished. The different controls on LexA most probably play important roles in this context: while LexA cleavage followed by proteolysis allows for a fast induction of the response, its autoregulation by negative feedback enables a quick shutoff.

The results presented here for the SOS response are in striking similarity to recent observations of the behavior of the p53-Mdm2 network in individual mammalian cells [23], both systems showing modulations in response to DNA damage. Remarkably, the bacterial system shows modulations that are more precise than the human system, a precision that is reminiscent of that found in developmental patterning [36] and in circadian clocks [37]. It would be interesting to test whether other stress response systems show similar properties and to investigate what may be the fitness advantage of such digital responses over analog ones. Such modulations are readily detected at the single-cell level, whereas slight timing differences smear them out on cell averages.

## Materials and Methods

### Strains and plasmids

The experiments were carried out in strain AB1157 *(argE3, his4, leuB6, proA2, thr1, ara14, galK2, lacY1, mtl1, xyl5, thi1, tsx33, rpsL31,* and *supE44)* [38] unless otherwise noted. Other strains used were WBY100 [39] (same as AB1157, but also *ΔumuDC*::cat), KY700 [40] *(Δ[pro-lac]*5 *thi ara met srlR::Tn10)* and LexA(Ind^−^) KY703 [40] (same as KY700, but also *lexA3 malE::Tn10*). The pGW2115 (K97A) plasmid carries the *umuDC* operon, with a point mutation Lys → Ala in position 97 of *umuD* [41]. To create the reporter plasmids, the promoter regions of the *recA,* *lexA, umuDC,* and *lacZ* genes were amplified by PCR from the DNA of the strain MG1655 and cloned into the plasmid pUA66 carrying the pSC101 origin of replication, using XhoI and BamHI upstream of a promoterless *GFPmut2* gene as described in [10,15]. Previously, it has been verified that the pSC101 average plasmid copy number per cell does not change after UV irradiation [10]. The addition of about 10 LexA binding sites due to the reporter plasmids [16] is expected to have a small effect on LexA occupation and on its regulation of the response because LexA has about 40 natural binding sites in the chromosome.

### Experimental setup

Experiments were carried out in a home-built inverted microscope, whose temperature was controlled and set to 37 °C. Images were acquired with an intensified camera (Videoscope International, Dulles, Virginia, United States; ICCD-350F), with integration times ranging from 0.1 to 4 s, and stored in a computer for later analysis. The samples were illuminated with an argon laser (488 nm) only during the time of integration of the camera to reduce photobleaching. Optical filters used were 480AF30 for excitation, 530DF30 for emission, and 505DRLP dichroic mirror (Omega Optical, Brattleboro, Vermont, United States). The incident power on the back aperture of the objective was approximately 5 μW, and the illumination area had a radius of approximately 50 μm. At these illumination level and exposure times, photobleaching was only appreciable after 4 h of experiment.

### Sample preparation

Cells were grown overnight in an LB medium and diluted into a fresh medium used in the experiments (M9 + 20 amino acids except tryptophan, 50 mg/l; thiamine 20 mg/l; thymine 20 mg/l; biotin 1 mg/l; glucose 0.4% v/v). After reaching midlog phase (OD_600_ = 0.25–0.4), cells were placed on a preheated agarose slab (experimental medium + 2% agarose) and incubated for 15 min at 37 °C. Cells were then irradiated in situ with UV light (wavelength 254 nm), using a low-pressure mercury germicidal lamp at levels between 10 and 50 J/m^2^ (20 J/m^2^ corresponds to an irradiation time of 12 s). After UV irradiation, bacteria were covered with a coverslip and monitored using the fluorescence microscope. The thin layer of agar allowed for an efficient supply of oxygen and nutrients to reach the cells during the experiment. Our irradiation procedure avoids any inhomogeneities due to the considerable UV absorption by the liquid media.

### Data analysis

The average intensity in a cell *I*(*t*) and its length *L*(*t*) were measured from fluorescence images after background subtraction, using MATLAB software (MathWorks, Natick, Massachusetts, United States). The product *I*(*t*)*L*(*t*) is proportional to the total amount of GFP within the cell at time *t.* Since GFP degradation and photobleaching are found to be negligible during the experiment, the time derivative of this amount corresponds to the GFP production rate, or PA: . The normalization by *L*(*t*) is analogous to the normalization by the optical density in measurements of cell populations [10]. To reduce noise in the PA calculation, which stems mainly from focus changes between consecutive images, we filtered *I*(*t*)*L*(*t*) with a digital Butterworth low-pass filter of order 4, and a cutoff frequency of 1/32 min^–1^. The filtration process was phase-preserving, to eliminate time delays. Filter parameters were chosen for maximal noise reduction while preserving the important dynamic features of the PA. The same filter parameters were used for all experiments. We verified that changing the filter's order or cutoff frequency by ± 50% did not significantly change the peaks' parameters. Values of PA were normalized by PA_0_, which was determined from the steady-state solution to the following equation for uninduced cells: *dI/dt* = PA_0_ **−** (l*n*2/<*T_D_*>)*I,* where <*T_D_*> is the mean doubling time of uninduced cells. *T_D_* is defined as the time it takes for a cell length, or for the sum of lengths of its daughter cells, to double, and it was extracted from the slope of exponential fits to *L*(*t*)*.*

The use of unstable GFP is not necessary since the high temporal resolution allows tracking of production changes. Control experiments in which high levels of GFP production were induced (fully induced *lac* promoter) neither showed modulations, nor affected the growth of the cells.

## Abbreviations

GFP: green fluorescent protein
PA: promoter activity
PA_0_: average PA of uninduced cells
PA/PA_0_: normalized PA
ssDNA: single-stranded DNA
UV: ultraviolet
